# The long noncoding RNAs PVT1 and uc002mbe.2 in sera provide a new supplementary method for hepatocellular carcinoma diagnosis

**DOI:** 10.1097/MD.0000000000004436

**Published:** 2016-08-07

**Authors:** Jinyu Yu, Junqing Han, Jian Zhang, Guanzhen Li, Hui Liu, Xianping Cui, Yantian Xu, Tao Li, Juan Liu, Chuanxi Wang

**Affiliations:** a Department of Oncology, Shandong Provincial Hospital Affiliated to Shandong University; b Department of Radiation Oncology, Shandong Cancer Hospital, Shandong Academy of Medical Sciences; c Department of Gastroenterology; d Department of Hepatobiliary Surgery; e Department of Infectious Disease, Shandong Provincial Hospital Affiliated to Shandong University, Jinan, Shandong Province, China.

**Keywords:** biomarker, hepatocellular carcinoma, long noncoding RNA, serum

## Abstract

Hepatocellular carcinoma (HCC) is the most common primary malignancy of the liver in adults worldwide. Several studies have demonstrated that long noncoding RNAs (lncRNAs) are involved in the development of various types of cancer, including HCC. These findings prompted us to examine the detectability of lncRNAs in blood samples from patients with HCC. In this study, we explored the expression levels of 31 cancer-related lncRNAs in sera from 71 HCC patients and 64 healthy individuals by reverse transcription and quantitative polymerase chain reaction (RT-qPCR). We found that 25 lncRNAs could be detected in the serum and that 7 had significantly different expression levels. A 2-lncRNA signature (PVT1 and uc002mbe.2) identified by stepwise regression showed potential as a diagnostic marker for HCC. The area under the receiver operating characteristic (ROC) curve was 0.764 (95% CI: 0.684–0.833). The sensitivity and specificity values of this serum 2-lncRNA signature for distinguishing HCC patients from the healthy group were 60.56% and 90.62%, respectively. The diagnostic ability of the combination of the serum 2-lncRNA signature with alpha-fetoprotein (AFP) was much greater than that of AFP alone. The expression levels of the 2 lncRNAs were associated with clinical parameters including tumor size, Barcelona Clinic Liver Cancer (BCLC) stage, and serum bilirubin.

## Abstract 2

Supplemental Digital Content is available in the text

## 1 Introduction

At present, hepatocellular carcinoma (HCC), which produces very high morbidity and mortality, is the most common primary malignancy of the liver in adults worldwide. HCC in China alone accounts for >50% of the cases worldwide due to the prevalence of hepatitis B virus (HBV)-induced hepatitis.^[1,2]^ Although many diagnostic and treatment methods are available for HCC (i.e., surgery, radiotherapy, chemotherapy, and biotherapy), HCC has a very poor prognosis, primarily due to metastasis, recurrence, and diagnostic delays.^[3,4]^ To date, alpha-fetoprotein (AFP) measurements have been widely used in clinical practice; however, a recent study showed that this assessment lacked adequate sensitivity and specificity for effective surveillance and diagnosis of HCC.^[4]^ Therefore, seeking effective early biomarkers for diagnosis and targets for therapy is essential for the diagnosis and treatment of HCC.

More than 90% of the human genome is transcribed into noncoding RNAs (ncRNAs). Long noncoding RNAs (lncRNAs) are longer than microRNAs (miRNAs), which are ∼22 nucleotides in length.^[5]^ With the development of high-resolution microarrays and massively parallel sequencing technology, numerous lncRNAs have been identified in humans. Recently, many investigators have reported that lncRNAs play critically important roles in the biological regulation, occurrence, and development of diseases.^[6]^ Notably, increasing evidence has indicated that many lncRNAs are involved in the carcinogenesis, development, and prognosis of HCC.^[7–9]^ What's more, some reported diagnostic marker for HCC could regulate lncRNAs that are also involved in HCC. For example, Cbx4/Pc2 regulates TUG1 and MALAT1/NEAT2.^[10]^ On one hand, Cbx4 has been reported as a diagnostic marker for HCC and promote HCC angiogenesis.^[11,12]^ On the other hand, TUG1 and MALAT1/NEAT2 are upregulated in HCC.^[13,14]^ Therefore, lncRNAs may be potential diagnostic markers and therapeutic targets for HCC in clinical practice.

Clinical biomarkers should be easily accessible and require noninvasive detection and sampling. LncRNAs are not significantly degraded when sera is treated with a prolonged room temperature incubation, multiple freeze-thaw cycles, a low or high pH solution, or RNase A digestion.^[15]^ An increasing number of circulating lncRNAs have been demonstrated to be dysregulated in plasma or serum, which demonstrates their high potential as powerful and noninvasive tumor markers.^[16,17]^ For example, circulating CUDR, LSINCT-5, and PTENP1 lncRNAs in sera can distinguish patients with gastric cancer from healthy controls, and the POU3F3 lncRNA in plasma is a novel biomarker for the diagnosis of esophageal squamous cell carcinoma. However, no systematic study has been performed to investigate the diagnostic value and clinicopathological influence of serum lncRNAs in HCC patients.

In this study, we selected 31 candidate cancer-related lncRNAs (supporting Table 1) that were identified in a previous study and tested their presence in sera. The fundamental aim was to determine whether these lncRNAs were detectable and altered in the sera of HCC patients compared to healthy controls. Then, we assessed the diagnostic values of the serum lncRNAs for HCC patients. Finally, the potential relationships between circulating lncRNA levels and the clinical features of HCC patients were investigated.

**Table 1:**
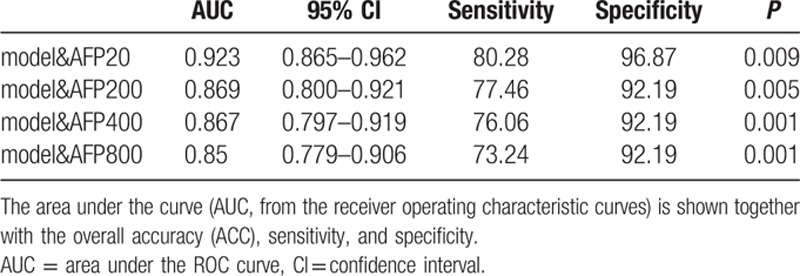
Performance of the predictive model in various combination sets.

## 2 Materials and methods

### 2.1 Study design

This study was divided into 4 phases.

Phase I (Gene selection): In this phase, 31 lncRNAs were measured in the sera of 11 pretreatment HCC patients and 10 healthy individuals to determine which lncRNAs were detectable.

Phase II (Marker selection): The lncRNAs that were detectable in phase I were evaluated in the sera of 31 pretreatment HCC patients and 31 healthy individuals to identify differentially expressed lncRNAs. Then, the lncRNAs with different expression levels between the HCC patients and healthy individuals were examined in the sera of an additional 40 HCC patients and 33 healthy individuals. The AFP level was evaluated in all the serum samples. The data obtained in this phase were used to construct the diagnostic model.

Phase III: The lncRNAs used to construct the diagnostic model were evaluated in the sera of 30 postoperative HCC patients whose preoperative serum samples were used in phase II.

Phase IV: We examined the correlations between the expression levels of the serum lncRNAs and the clinical parameters of all 71 HCC patients included in phase II.

### 2.2 Collection of serum samples and clinical data

All serum samples were randomly selected at Provincial Hospital Affiliated to Shandong University. The diagnosis of HCC was histopathologically or clinically confirmed. None of the patients received anticancer treatment prior to hospitalization. Healthy individuals referred to individuals with no liver disease or any type of malignant tumor, who were defined as the control group. Sera were collected from the peripheral blood of all subjects and separated on individual gels. The serum samples were stored in RNase and DNase-free tubes (Axygen, Tewksbury MA) at –80 °C prior to total RNA extraction.

The data obtained from all the subjects’ medical records included age, gender, alcohol consumption, HBV infection status, results of the liver function test, AFP level, and HCC features such as tumor size, location, histology, depth of invasion, the status of lymphatic metastasis, vascular invasion, intrahepatic/distant metastasis, and the Child–Pugh class. The HCC clinical stage was determined based on the tumor node metastasis (TNM) classification system and the BCLC classification system.^[18,19]^ The written informed consent was obtained from all participants. All experimental protocols were approved by Ethical Committee of Provincial Hospital Affiliated to Shandong University and was performed according to the Declaration of Helsinki.

### 2.3 RNA isolation, reverse transcription (RT), and quantitative PCR (qPCR)

Total RNA was extracted from the serum samples using a Blood Total RNA Isolation Kit (RP4002, BioTeke Corporation, Beijing, China) according to the manufacturer's protocol. RT and qPCR kits were utilized to evaluate the expression of lncRNAs in the serum samples. The 20 μL RT reactions were performed using a PrimeScript RT Reagent Kit (Takara, Dalian, China) consisting of 10 μL of total RNA solution, 1 μL of PrimeScript RT Enzyme Mix I, 1 μL of the RT Primer Mix, 4 μL of 5 × PrimeScript Buffer, and 4 μL of RNase-free dH_2_O. The mixture was incubated at 37 C for 15 minutes and 85 C for 5 seconds and then maintained at 4 C. The qPCR was performed using SYBR Premix Ex Taq (Takara, Dalian, China) with 2 μL of the RT products mixed with 10 μL of SYBR Premix Ex Taq II, 0.4 μL of the gene-specific forward and reverse primers (10 μM), and 7.2 μL of nuclease-free water. The primers used in this study are summarized in supporting Table 1. All reactions were performed with the Roche LightCycler 480II (Roche, Switzerland) using the following conditions: 95 C for 30 seconds, followed by 45 cycles of 95 C for 5 seconds and 60 C for 30 seconds. The amplification of the appropriate product was confirmed by melting curve analysis. Glyceraldehyde-3-phosphate dehydrogenase (GAPDH) was used as an endogenous control to normalize the data because it is stably expressed in plasma.^[15]^ The sequences of the primers used for the GAPDH amplification were 5′-CCGGGAAACTGTGGCGTGATGG-3′ (forward) and 5′-AGGTGGAGGAGTGGGTGTCGCTGTT-3′ (reverse). The reactions were performed in duplicate or triplicate according to the acquired volume of the samples. Samples with a CT > 40 were considered negative. Relative expression of the target lncRNAs was calculated using the 2^−△△CT^ method.

### 2.4 Serum AFP determination

The serum AFP level was measured using the Roche Cobas 8000 machine (Roche, Switzerland).

### 2.5 Statistical analysis

All statistical analyses were performed using the SPSS 17.0 software (SPSS Inc.), with the exception of the cross validation, which was performed with SAS9.4 software (SAS Institute Inc, Carrey, NC). Expression differences in lncRNAs in the sera of patients and healthy individuals were determined by a 2-tailed *t* test. Multivariate classification models were constructed to determine the combination of the selected lncRNAs that had the greatest predictive ability for cancer. Receiver operating characteristic (ROC) curves were plotted, and the area under the ROC curve (AUC) was calculated to assess the specificity and sensitivity of predicting HCC patients and controls. Cross-validation was used to test the diagnostic model. ANOVA was used for the analysis of the correlation between the lncRNA expression levels and clinical parameters when >2 groups were present; otherwise, a *t* test was used. The Wilcoxon signed-rank test was used to compare the lncRNA expression levels between the sera of preoperative and postoperative HCC patients.

## 3 RESULTS

### 3.1 Screening for detectable lncRNAs in the sera of HCC patients and healthy individuals

To explore the potential use of serum lncRNAs as biomarkers for HCC, first we determined which lncRNAs could be detected by RT-qPCR in pretreatment sera from HCC patients and healthy individuals. In the gene selection phase, 25 of 31 lncRNAs (supporting information table S1) were detectable in the serum samples from 11 patients and 10 healthy individuals. The lncRNAs that could not be detected were excluded in the next phase.

### 3.2 Constructing the diagnostic model

**Figure 1:**
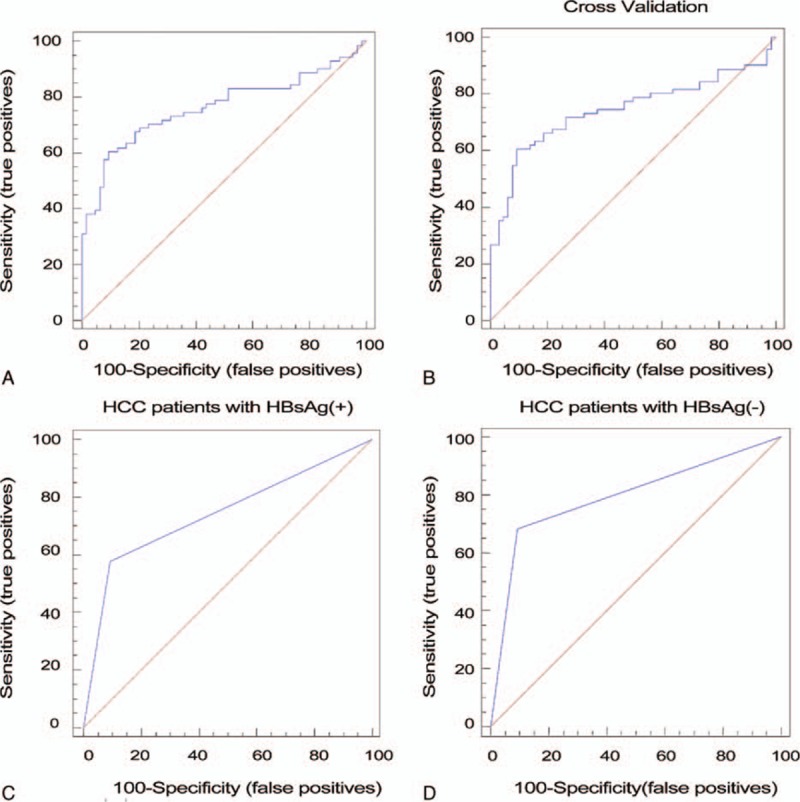
The serum 2-lncRNA diagnostic model. The relative expression level of a panel consisting of 2 lncRNAs was calculated using the regression equation generated by stepwise regression analysis. (A) Receiver operating characteristic (ROC) curve of the serum 2-lncRNA diagnostic model used in phase II to discriminate HCC patients from healthy subjects. (B) The ROC curve of the 10-fold cross validation of the serum 2-lncRNA signature in the phase II samples. (C) The ROC curve of the diagnostic model used to discriminate HBsAg-positive patients from healthy individuals. (D) The ROC curve of the diagnostic model used to discriminate HBsAg-negative patients from healthy individuals. HBsAg = hepatitis B surface antigen, HCC = hepatocellular carcinoma, lncRNA = long noncoding RNA, ROC = receiver operating characteristic.

In phase II, the 25 lncRNAs were analyzed in serum samples from 31 pretreatment HCC patients and 31 healthy individuals. Eight lncRNAs showed significantly different expression levels (up- or downregulated) between the 2 groups (supporting information table S1). ATB, EBIC, hDREH, PVT1, SRHC, RERT, and uc002mbe.2 were significantly upregulated, and CUDR was significantly downregulated. RERT was excluded from the remaining experiments because it was detected in <50% of the samples. The 7 remaining lncRNAs were measured in the sera of an additional 40 HCC patients and 33 healthy individuals. All the data collected in phase II were used to construct a stepwise selection model. The model revealed that the combination of PVT1 and uc002mbe.2 showed the greatest predictive ability for HCC (supporting information table S2) with an AUC of 0.764 (95% CI: 0.684–0.833) (Fig. 1A) and a 10-fold cross-validation of 0.744 (95% CI: 0.662–0.815) (Fig. 1B).

Because many of the HCC patients were infected with HBV, we examined the AUC of the patients who tested positive or negative for hepatitis B surface antigen (HBsAg). We found that the AUC of the HBsAg-positive patients was 0.742 (95% CI: 0.652–0.818) (Fig. 1C), and the AUC of the HBsAg-negative patients was 0.795 (95% CI: 0.692–0.876) (Fig. 1D). These results suggest that the combination of the 2 lncRNAs can distinguish HCC patients from healthy individuals regardless of whether the patient is infected with HBV.

### 3.3 Comparing and combining the diagnostic model with the AFP level

**Figure 2:**
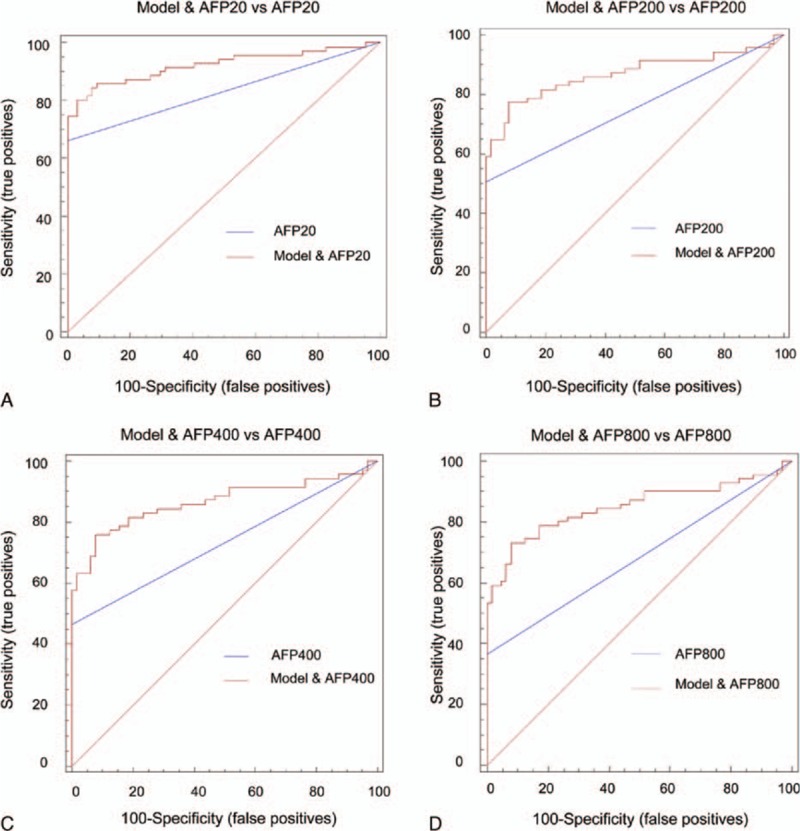
The ROC curve of AFP with different cut-off values and the ROC curve of the model combined with the AFP level. (A) The cut-off value of AFP is 20 μg/L. (B) The cut-off value of AFP is 200 μg/L. (C) The cut-off value of AFP is 400 μg/L. (D) The cut-off value of AFP is 800 μg/L. AFP = alpha-fetoprotein, ROC = receiver operating characteristic.

The AFP level was analyzed in the serum samples, and the AUC of AFP was calculated. We compared the AUC values of the 2 selected lncRNAs to the AFP AUC value. The predictive ability of the lncRNA combination was equal to that of AFP for discriminating HCC patients from controls when the AFP cut-off value was 20 μg/L, 200 μg/L, 400 μg/L, or 800 μg/L (Fig. 2A–D, supporting information table S3). In this study, our diagnostic model showed higher sensitivity than the AFP level. We combined the model with the AFP level and discovered that they were complementary. When the cut-off values of AFP were 20 μg/L, 200 μg/L, 400 μg/L, and 800 μg/L, the AUCs of the combination of the lncRNAs and AFP were 0.923, 0.869, 0.867, and 0.85, respectively. The predictive ability was remarkably higher than that of the AFP level alone (Fig. 2A–D, Table 1).

### 3.4 Comparison of lncRNA expression levels in the sera of preoperative and postoperative HCC patients

**Figure 3:**
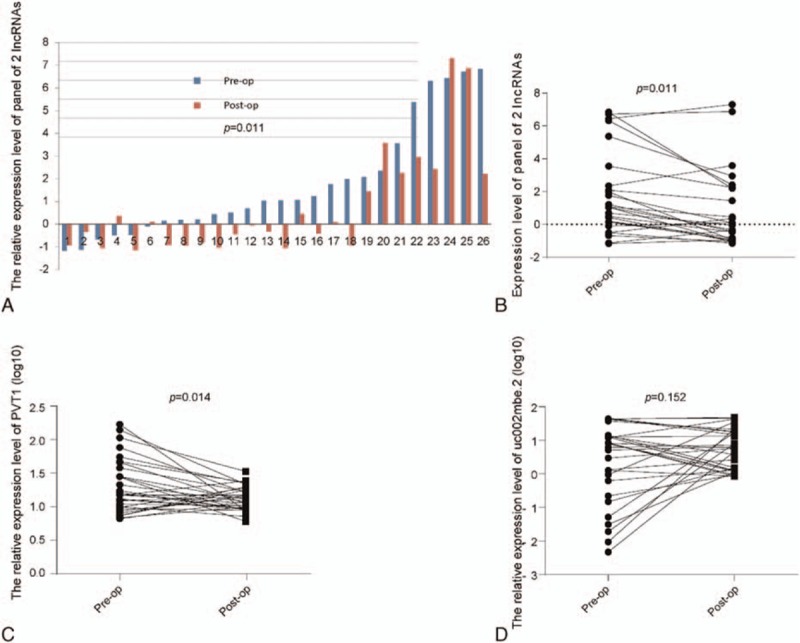
The lncRNA expression levels were examined by real-time PCR in postoperative and preoperative samples and analyzed by the Wilcoxon signed-rank test. (A, B) The expression levels of the panel consisting of 2 lncRNAs in the preoperative and postoperative serum samples. The 5% of the samples with the highest and lowest difference values between the preoperative and postoperative measurements were not included in the figures. (C) The expression level of PVT1 in the preoperative and postoperative serum samples. (D) The expression level of uc002mbe.2 in the preoperative and postoperative serum samples. lncRNA = long noncoding RNA, qPCR = polymerase chain reaction.

We analyzed the expression levels of the 2 lncRNAs in a cohort comprised of 30 postoperative HCC patients whose preoperative sera were used in phase II to determine whether the relative expression levels of the 2 lncRNAs had changed. We found that the expression levels of the 2 lncRNAs had significantly decreased after the patients underwent the operation (Fig. 3A and B). When we analyzed the 2 lncRNAs respectively we found that only expression of PVT1 decreased after surgery.

### 3.5 Correlation between marker expression and clinical features

The expression levels of many lncRNAs in tissues or sera have been associated with the clinical features of cancer. Therefore, we examined the correlation between the expression levels of the panel containing the 2 lncRNAs and several clinical parameters. A significant association was observed between the 2 lncRNAs and tumor size, BCLC stage, and the serum bilirubin level (*P* < 0.05, Table 2). No significant association was found between the 2 lncRNAs and age, gender, alcohol use, smoking status, AFP level, HBsAg, microvascular invasion, macrovascular metastasis, lymphatic metastasis, portal hypertension, serum albumin, Child–Pugh grade, or TNM stage (*P* > 0.05, Table 2).

**Table 2:**
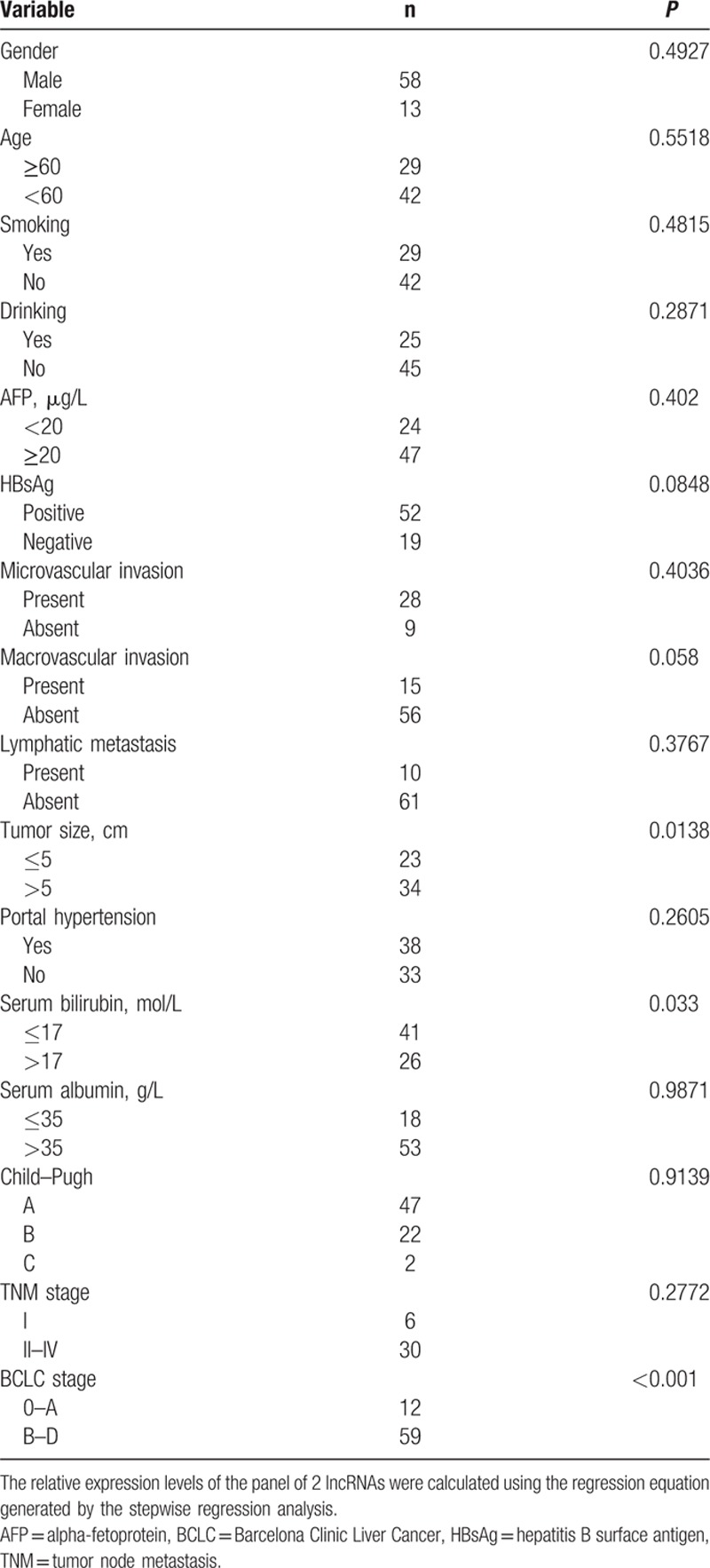
Correlations between serum PVT1 and uc002mbepanel expression levels and clinical parameters.

## 4 DISCUSSION

Biomarkers are a vitally important part of clinical diagnosis and treatment. Many studies have focused on identifying effective biomarkers (especially blood biomarkers) because they are easy to obtain, do not require invasive methods, and have a lower economic burden. To date, the serum concentration of AFP is the most frequently used marker for the diagnosis of HCC.^[4]^ A large systematic review that contained 5 studies showed the following prognostic values of AFP using a cut-off value of > 20 μg/L: sensitivity of 41% to 65%, specificity of 80% to 94%, negative likelihood ratio of 0.4 to 0.6, and a positive likelihood ratio of 3.1 to 6.8.^[4]^ However, increasing data have revealed that the sensitivity and specificity of AFP are not sufficient for effective diagnosis. Therefore, there is a great need for more effective biomarkers for HCC.

Recently, lncRNAs have attracted attention due to their role in disease generation and development.^[7,20,21]^ However, most studies have focused on the correlation between lncRNAs and tumor tissues and cells. Recent studies have revealed that lncRNAs are stable in human serum and can be readily detected by qRT-PCR.^[22]^ Thus, lncRNAs show great potential to serve as biomarkers for cancer diagnosis. For example, lncRNA H19 in circulation was proposed as a marker for the diagnosis of gastric cancer with an AUC of 0.64.^[23]^ However, the sensitivity and specificity of biomarkers based on a single tumor-specific lncRNA are generally poor, and adding the analysis of other related lncRNAs may increase the diagnostic value. For example, the circulating CUDR, LSINCT-5, and PTENP1 lncRNAs in sera could distinguish patients with gastric cancer from healthy controls with an AUC of up to 0.829, which was much higher than the predictive ability reported for CEA and CA19-9.^[24]^ To date, there has only been 1 report concerning the diagnosis of HCC; however, this report used a single tumor-specific lncRNA, and the systematic study was deficient.^[16]^

**Figure 4:**
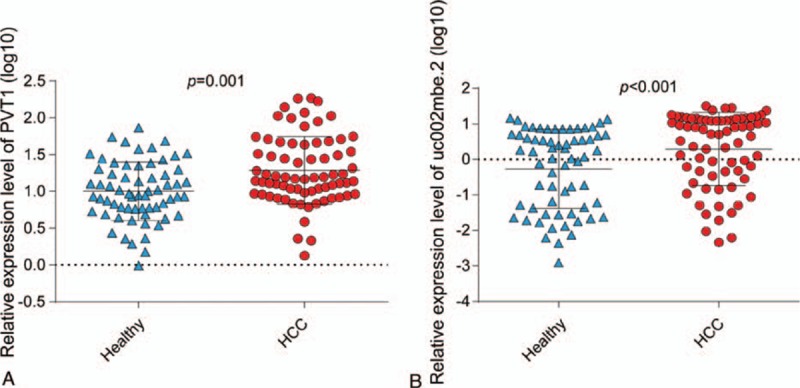
The expression levels of PVT1 (A) and uc002mbe.2 (B) were significantly different in the sera of HCC patients compared with the levels in healthy subjects, as determined by RT-qPCR and analyzed by a *t* test. HCC = hepatocellular carcinoma, RT-qPCR = quantitative polymerase chain reaction.

In this study, we found that 25 of 31 lncRNAs could be detected in HCC patients and healthy individuals, indicating that some lncRNAs might have extreme tissue specificity. Then, using qRT-PCR, we demonstrated that the expression levels of 7 cancer-associated lncRNAs varied significantly between the sera of HCC patients and healthy controls (supporting information table S1). Five of these lncRNAs have been confirmed to be associated with HCC tissues.^[8,9,25–27]^ We screened the expression levels of the 7 lncRNAs in the sera of 71 HCC patients and 64 healthy individuals. Stepwise model selection showed that the combination of PVT1 and uc002mbe.2 provided the greatest ability to discriminate between HCC patients and healthy individuals with an AUC of 0.764 (95% CI: 0.684–0.833). The sensitivity and specificity were 60.56% and 90.62%, respectively (Fig. 1A, supporting information table S2). The lncRNAs PVT1 and uc002mbe.2 were upregulated in the sera of HCC patients compared with healthy controls (Fig. 4), which shown to have significantly differential expression patterns between HCC tissues and normal liver tissues. Therefore, lncRNAs in the sera may have disease specificity and diagnostic potential.

Four AFP cut-off values are frequently used in clinical practice: 20 μg/L, 200 μg/L, 400 μg/L, and 800 μg/L. The AUCs of AFP for our samples at the 4 cut-off values were 0.831, 0.754, 0.732, and 0.683, respectively (Fig. 2A–D, supporting information table S3). In our study, all the control samples consisted of sera collected from individuals without liver disease and with normal AFP levels. Thus, the specificity reached 100%. In clinical practice, the diagnostic ability may be lower than our result. Some studies have reported that the concentration of serum AFP was increased in patients with acute or chronic viral hepatitis and in patients with chronic hepatic disease without HCC.^[28,29]^ Additionally, the serum AFP level was reported to be normal in up to 40% of patients with small HCCs.^[30]^ Our data showed that our model and AFP were complementary and produced higher sensitivity. When we combined our model with the AFP level we found that the predictive ability to discriminate HCC patients from healthy controls was much greater than the predictive ability of the AFP level alone (Fig. 2A–D, Table 1). In other words, our model is a good supplement for HCC diagnosis. Moreover, the diagnostic ability was not influenced by HBsAg. Indeed, the diagnostic model was applicable for both groups regardless of whether the patient was infected with HBV (Fig. 1C and D). This finding may indicate that the 2 lncRNAs are involved in the process of HCC generation and development but not in the HBV-induced development of HCC.

After the operation, the expression level of the panel of 2 lncRNAs was significantly decreased compared to the preoperative samples, which mainly due to the decrease of PVT1. This finding may indicate that the serum expression levels of lncRNAs are related to the HCC tumor. Determining how the tumor influences the lncRNA expression level requires further exploration. All tumor features (i.e., tumor size, pathological pattern, and location) may have an effect on the serum expression levels.

Finally, we analyzed the correlation between the expression levels of the 2 lncRNAs and various clinical characteristics. The tumor tissue expression level of PVT1 has been reported to be associated with the tumor size. Our data showed that the combination of lncRNAs was correlated with the tumor size,^[27]^ BCLC stage, and serum bilirubin level. These results may explain why the expression level was reduced after the operation and indicated that the lncRNAs may be useful for monitoring tumor progression and recurrence. When the tumor was resected, the tumor size was reduced, and thus the expression levels were decreased. The serum bilirubin level and BCLC stage may contribute to the function of the 2 lncRNAs.

Functional studies of lncRNAs in tumor tissues are helpful for evaluating serum lncRNAs as indicators of various types of cancer. lncRNA-PVT1 has received much attention among oncogenic lncRNAs because it is involved in many types of tumor tissues, such as breast cancer, ovarian cancer, lung cancer, and gastric cancer.^[31–34]^ This lncRNA is upregulated in gastric cancer, lung cancer, pancreatic cancer, and other cancers and is thought to play a role in drug resistance and tumor characteristics.^[35]^ lncRNA-mPvt1 was first identified in mice, where it was upregulated in early liver tissues and HCC cell lines. The human ortholog of lncRNA-mPvt1 is lncRNA-PVT1, which is significantly upregulated in HCC tissues.^[27,36]^ LncRNA-PVT1 can promote HCC cell proliferation in vitro and in vivo, regulate the expression of cell cycle genes, predicts HCC recurrence following liver transplantation; moreover, it is involved in the pathway associated with the development of HCC.^[27,36]^ The change in its expression level between sera from patients and healthy controls was consistent with the change between HCC tumor tissue and peritumoural tissue. To date, lncRNA uc002mbe.2 has only been studied in HCC tissues.^[25]^ It has been reported to be associated with the trichostatin-mediated apoptosis of HCC cells and was downregulated in HCC tissues, which is inconsistent with the serum expression level change. This phenomenon was also reported in gastric cancer, where the CUDR lncRNA was upregulated in gastric cancer tissue but downregulated in patient sera.^[24]^ In our study, the expression of CUDR was also downregulated in patient sera compared to sera from the healthy group; however, there have been no reports about the detection of this lncRNA in HCC tissues to date.

This inconsistency led us to speculate about the source and transfer mechanism of circulating lncRNAs. This inconsistency between tissue and serum expression might be caused by the mechanism of secretion of circulating RNA. Some lncRNAs have been reported to be secreted into cell culture medium at measurable levels.^[24]^ However, the levels of lncRNAs with intermediate expression differ in different cell culture media and are not as stable as that of an internal reference. This result might indicate that different cells secrete lncRNAs at different levels, and this phenomenon might be regulated by a complex system. The circulating uc002mbe.2 lncRNA might be discharged by tumor cells in a more redundant manner than in normal cells. The definite process by which lncRNAs enter into circulation and are transferred needs to be elucidated in the future. An absence of a significant correlation has been reported between the expression levels of lncRNAs in plasma and tumor tissues, but the mechanisms underlying this phenomenon are unknown.^[23]^

An increasing number of studies have revealed that lncRNAs are stable in circulation.^[15,23,24,37]^ Circulating lncRNAs may be protected by extracellular vesicles (EVs), including apoptotic bodies, microvesicles, and exosomes, and may represent a form of communication between cells, tissues, and organs.^[38,39]^ lncRNAs may also form complexes with proteins that are similar to those observed for circulating miRNAs or with miRNAs in tissues; in turn, these complexes may contribute to their functions and effects.^[40–42]^ The stability and existence of latent functions of serum lncRNAs contributes to their potential as biomarkers or therapeutic targets.^[43,44]^ Future studies should focus on the source and existence of the selected markers and their functions in the serum.

Sera lncRNAs have good potential to serve as biomarkers for disease diagnosis due to their stability, relationship with the disease, and easy acquisition. In this study, we analyzed 31 lncRNAs in sera from HCC patients. As more lncRNAs are identified, analyses will be used to determine better biomarkers for different diseases. Serum lncRNAs can be screened for early diagnosis, to evaluate surgical or nonsurgical therapeutic efficiency, to monitor recurrence during the follow-up period, and to provide new therapeutic targets.

In conclusion, our data showed that the combination of the 2 lncRNAs in sera provided a new supplementary method for HCC diagnosis. This achievement may improve the diagnostic ability of biomarkers for HCC in clinical practice and provide directions for future studies of biomarkers, such as their functions, clinical values, and structures.

## Supplementary Material

Supplemental Digital Content
