# Supplementary material for: The long noncoding RNAs PVT1 and uc002mbe.2 in sera provide a new supplementary method for hepatocellular carcinoma diagnosis
Source: Medicine (Baltimore). 2016 Aug 7;95(31):e4436. doi: 10.1097/MD.0000000000004436 (PMC4979822; doi:10.1097/MD.0000000000004436)
Supplement: Supplemental Digital Content [file medi-95-e4436-s001.doc]

| **Gene** | **Primer sequence** | **Phase** Ⅰ | | **Phase Ⅱ p value 1** | | **Phase Ⅱ** |  | |
| --- | --- | --- | --- | --- | --- | --- | --- | --- |
| HOTAIR | Forward:5'-GGTAGAAAAAGCAACCACGAAGC-3' | P | | 0.1359 | |  |  | |
|  | Reverse:5'-ACATAAACCTCTGTCTGTGAGTGCC-3' |  |  |  |  |  |  | |
| HULC | Forward:5'-ATCTGCAAGCCAGGAAGAGTC-3' | N | |  | |  |  | |
|  | Reverse:5'-CTTGCTTGATGCTTTGGTCTGT-3' |  |  |  | |  |  | |
| H19 | Forward:5'-TGCTGCACTTTACAACCACTG-3' | P | | 0.9461 | |  |  | |
|  | Reverse:5'-ATGGTGTCTTTGATGTTGGGC-3' |  |  |  |  |  |  | |
| PRNCR1 | Forward:5'-CCAGGGGGAAACACACAG-3' | P | | 0.6763 | |  |  | |
|  | Reverse:5'-AAATGGCAGTTTCCTTCAATG-3' |  |  |  |  |  |  | |
| LOC285194 | Forward:5'-TGTGCCTGTTTGACCTCTGA-3' | P | | 0.782 | |  |  | |
|  | Reverse:5'-AGGAAGGATAAAAGACCGACCA-3' |  |  |  |  |  |  | |
| PCGEM1 | Forward:5'-TGCCTCAGCCTCCCAAGTAAC-3' | P | | 0.3052 | |  |  | |
|  | Reverse:5'-GGCCAAAATAAAACCAAACAT-3' |  |  |  |  |  |  | |
| BIC | Forward1:5'-TCAAGAACAACCTACCAGAGACCTT-3' | N | |  | |  |  | |
|  | Reverse1:5'-TCCTGGTTTGTGCCACCAT-3' |  |  |  | |  |  | |
|  | Forward2:5'-ACCAGAGACCTTACCTGTCACCTT-3' |  |  |  | |  |  | |
|  | Reverse2:5'-GGCATAAAGAATTTAAACCACAGATTT-3' |  |  |  | |  |  | |
| UCA1 | Forward:5'-CTCTCCATTGGGTTCACCATTC-3' | P | | 0.5392 | |  |  | |
|  | Reverse:5'-GCGGCAGGTCTTAAGAGATGAG-3' |  |  |  |  |  |  | |
| CUDR | Forward:5'-GCACCCTAGACCCGAAA-3' | P | | 0.0096 | | 0.037 |  | |
|  | Reverse:5'-GCCACCTGGACGGATAT-3' |  |  |  |  |  |  | |
| uc.338 | Forward:5'-AGCGACAGTGCGAGCTTT-3' | P | | 0.4479 | |  |  | |
|  | Reverse:5'-GGAAGGATTGAGTGAGCCTT-3' |  |  |  |  |  |  | |
| MALAT1 | Forward:5'-AACGCAGACGAAAATGGAAAGA-3' | N | |  | |  |  | |
|  | Reverse:5'-CCTTCTAACTTCTGCACCACCAGA-3' |  |  |  | |  |  | |
| SRA | Forward:5'-AGGAACGCGGCTGGAACGA-3' | N | |  | |  |  | |
|  | Reverse:5'-AGTCTGGGGAACCGAGGAT-3' |  |  |  | |  |  | |
| PTENP1 | Forward:5'-AGTCACCTGTTAAGAAAATGAGAAGACAAA-3' | P | | 0.2052 | |  |  | |
|  | Reverse:5'-CTGTCCCTTATCAGATACATGACTTTCAA-3' |  |  |  |  |  |  | |
| Nbla10727 | Forward:5'-CAGTCAGCCTCAGTTTCCAA-3' | P | | 0.5251 | |  |  | |
|  | Reverse:5'-AGGCAGGGCTGTGCTGAT-3' |  |  |  |  |  |  | |
| Nbla12061 | Forward:5'-ATGTTAGCTCCCAGCGATGC-3' | P | | 0.9866 | |  |  | |
|  | Reverse:5'-CTAACTGCCAAAAGGTTTTCC-3' |  |  |  |  |  |  | |
| LSINCT5 | Forward:5'-TTCGGCAAGCTCCTTTTCTA-3' | P | | 0.109 | |  |  | |
|  | Reverse:5'-GCCAAGTCCCAAAAAGTTCT-3' |  |  |  |  |  |  | |
| LOC554202 | Forward1:5'-TTTTTCTATCACTGCCTTTTCACA-3' | N | |  | |  |  | |
|  | Reverse1:5'-GCCCCCAACTCTATTTACCAA-3' |  |  |  | |  |  | |
|  | Forward2:5'-TAGGGCTGCCAGTAGAGGGAAGAG-3' |  |  |  | |  |  | |
|  | Reverse2:5'-GCAAGCAGGCCAACCAACAAG-3' |  |  |  | |  |  | |
| PlncRNA-1 | Forward:5'-AGTAGTTGCTTGTCCTAT-3' | N | |  | |  |  | |
|  | Reverse:5'-AAGTCAGTAAGTCCTAAGT-3' |  |  |  | |  |  | |
| HEIH | Forward:5'-CCTCTTGTGCCCCTTTCTT-3' | P | | 0.1727 | |  |  | |
|  | Reverse:5'-ATGGCTTCTCGCATCCTAT-3' |  |  |  |  |  |  | |
| ATB | Forward:5'-TCTGGCTGAGGCTGGTTGAC-3' | P | | 0.0062 | | <0.01 |  | |
|  | Reverse:5'-ATCTCTGGGTGCTGGTGAAGG-3' |  |  |  |  |  |  | |
| PCNA-AS1 | Forward:5'-CTTCAAATACTAGCGCCAAGGTATC-3' | P | | 0.3821 | |  |  | |
|  | Reverse:5'-TTTTTTTCGCAACGCGGCGCAGGGT-3' |  |  |  |  |  |  | |
| SRHC | Forward:5'-GGAGGACCAGCTGCTGTAAG-3' | P | | 0.0007 | | 0.016 |  | |
|  | Reverse:5'-CCAGAGAGGTGGTGGTGTTT-3' |  |  |  |  |  |  | |
| hDREH | Forward:5'-GCTAACGAACAAAGCCAGA-3' | P | | 0.0255 | | 0.015 |  | |
|  | Reverse:5'-CCCTATTCTCATGCAAGGA-3' |  |  |  |  |  |  | |
| EBIC | Forward:5'-GACTGAATGGACAAGTGGATCTTC-3' | P | | 0.0022 | | <0.01 |  | |
|  | Reverse:5'-GGAGTTCTTCTTGACCCTCTTGTAG-3' |  |  |  |  |  |  | |
| hLALR1 | Forward:5'-ACGGGTGCGGGTTTAGG-3' | P | | 0.3636 | |  |  | |
|  | Reverse:5'-TCCAGGGCCGACTCCAT-3' |  |  |  |  |  |  | |
| PVT1 | Forward:5'-AAAACGGCAGCAGGAAATGT-3' | P | | 0.0025 | | <0.01 |  | |
|  | Reverse:5'-ATTCCCATAGAAGGGGCAGG-3' |  |  |  |  |  |  | |
| LET | Forward:5'-GTTGTTGTTGCATTGGGGT-3' | P | | 0.7229 | |  |  | |
|  | Reverse:5'-AAGATGGAGAGTGGAGCCT-3' |  |  |  |  |  |  | |
| HOTTIP | Forward:5'-TTCTGCAGTGAGACCACAGG-3' | P | | 0.1508 | |  |  | |
|  | Reverse:5'-AACAGTGTGGACAGGGAAGG-3' |  |  |  |  |  |  | |
| MEG3 | Forward:5'-CTGCCCATCTACACCTCACG-3' | P | | 0.6276 | |  |  | |
|  | Reverse:5'-CTCTCCGCCGTCTGCGCTAGGGGCT-3' |  |  |  |  |  |  | |
| RERT | Forward:5'-CGGAGAGGATGGGCTCTGGCATT-3' | P | | 0.0387 | |  |  | |
|  | Reverse:5'-AGGGACCCTTCAGGGTGGCTG-3' |  |  |  |  |  |  | |
| uc002mbe.2 | Forward:5'-TTGTCTCCCTGTTACACTGTGA-3' | P | | 0.047 | | <0.01 |  | |
|  | Reverse:5'-GGTTTATTCTTTGATGCCTTTAT-3' |  |  |  |  |  |  | |
| N: negative |  |  | |  | |  |  | |
| P: positive |  |  | |  | |  |  | |
| Phase Ⅱ p value 1: Tha data included 31 pre-treatment HCC patients and 31 healthy individuals in phase Ⅱ. | | | | | | |  | |
| Phase Ⅱ: Tha data included all the subjects in phase Ⅱ. | | |  | |  | | |  |

Table S2. Multivariate stepwise logistic regression analysis* of the 2 lncRNAs in the sera of patients with hepatocellular carcinoma.

| lncRNA | Regression coefficient | OR (95% CI) | *p* |
| --- | --- | --- | --- |
| hPVT1 | 0.0363 | 1.037(1.014-1.060) | 0.0013 |
| uc002mbe.2 | 0.1749 | 1.191(1.098-1.293) | <0.001 |
|  |  |  |  |

*Stepwise selection was used to determine the diagnostic values of serum lncRNAs for hepatocellular carcinoma (HCC). Among the 7 lncRNAs that were differentially expressed between the HCC and healthy groups, the combination of PVT1 and uc002mbe.2 was chosen as the strongest panel for the diagnosis of HCC. The other lncRNAs, including ATB, EBIC, hDREH, SRHC, and CUDR, were excluded by a stepwise procedure.

The regression equation: The relative expression level of a panel of 2 lncRNAs= -1.5221+0.0363×PVT1+0.1749×uc002mbe.2.

CI: confidence interval. OR: odds ratio.

Performance of the predictive model in various sets

|  | AUC | 95%CI | sensitivity | specificity | *p* |
| --- | --- | --- | --- | --- | --- |
| Model | 0.764 | 0.684 to 0.833 | 60.56 | 90.62 |  |
| AFP20 | 0.831 | 0.757 to 0.890 | 66.2 | 100 | 0.209 |
| AFP200 | 0.754 | 0.672 to 0.824 | 50.7 | 100 | 0.845 |
| AFP400 | 0.732 | 0.649 to 0.805 | 46.48 | 100 | 0.574 |
| AFP800 | 0.683 | 0.598 to 0.760 | 36.62 | 100 | 0.174 |

The area under the curve (AUC, from the receiver operating characteristic curves) is shown together sensitivity and specificity. A value of P<0.05 was considered the comparison of the diagnostic of the model with other set statistically significant.

CI: confidence interval.
